# Human introns contain conserved tissue-specific cryptic poison exons

**DOI:** 10.1093/nargab/lqae163

**Published:** 2024-12-11

**Authors:** Sergey Margasyuk, Antonina Kuznetsova, Lev Zavileyskiy, Maria Vlasenok, Dmitry Skvortsov, Dmitri D Pervouchine

**Affiliations:** Center for Molecular and Cellular Biology, Skolkovo Institute of Science and Technology, Bolshoy Bulvar, 30, 121205, Moscow, Russia; Center for Molecular and Cellular Biology, Skolkovo Institute of Science and Technology, Bolshoy Bulvar, 30, 121205, Moscow, Russia; Center for Molecular and Cellular Biology, Skolkovo Institute of Science and Technology, Bolshoy Bulvar, 30, 121205, Moscow, Russia; Center for Molecular and Cellular Biology, Skolkovo Institute of Science and Technology, Bolshoy Bulvar, 30, 121205, Moscow, Russia; Center for Molecular and Cellular Biology, Skolkovo Institute of Science and Technology, Bolshoy Bulvar, 30, 121205, Moscow, Russia; Faculty of Chemistry, Moscow State University, Ul Kolmogorova, 1, 119991, Moscow, Russia; Center for Molecular and Cellular Biology, Skolkovo Institute of Science and Technology, Bolshoy Bulvar, 30, 121205, Moscow, Russia

## Abstract

Eukaryotic cells express a large number of transcripts from a single gene due to alternative splicing. Despite hundreds of thousands of splice isoforms being annotated in databases, it has been reported that the current exon catalogs remain incomplete. At the same time, introns of human protein-coding (PC) genes contain a large number of evolutionarily conserved elements with unknown function. Here, we explore the possibility that some of them represent cryptic exons that are expressed in rare conditions. We identified a group of cryptic exons that are similar to the annotated exons in terms of evolutionary conservation and RNA-seq read coverage in the Genotype-Tissue Expression dataset. Most of them were poison, i.e. generated an nonsense-mediated decay (NMD) isoform upon inclusion, and many showed signs of tissue-specific and cancer-specific expression and regulation. We performed RNA-seq in A549 cell line treated with cycloheximide to inactivate NMD and confirmed using quantitative polymerase chain reaction that seven of eight exons tested are, indeed, expressed. This study shows that introns of human PC genes contain cryptic poison exons, which reside in conserved intronic regions and remain not fully annotated due to insufficient representation in RNA-seq libraries.

## Introduction

Evolutionary conservation is widely accepted as a proxy for biological function (1–3). Out of 3.1 billion base pairs in the human genome, ∼1.1 billion (38%) are located in protein-coding (PC) genes, of which only 25 million make up coding exons, while the rest comprise introns and untranslated regions (UTRs) (4). Despite introns being spliced and removed, ∼50 million base pairs in them are conserved across vertebrates (5), which makes it reasonable to ask about the function of these sequences. In this work, we explore the possibility that some of the conserved intronic elements represent cryptic exons.

To date, the most widely-used transcript reference sets are GENCODE and RefSeq, which provide manually curated and automatically generated transcript sets that incorporate evidence from RNA-seq data (6,7). Other comprehensive catalogs such as CHESS (8) and VastDB (9) infer expressed transcripts from large panels of human and vertebrate tissue transcriptomes. However, growing evidence suggests that these catalogs remain incomplete because of systematic bias against splice isoforms with low abundance (10). Despite recent incorporation of long-read sequencing data into these databases (6,11), many transcript isoforms that have low expression levels or appear in rare biological conditions are still missing (12–14).

One of the reasons why the expression level may be low is that the transcript is targeted by the nonsense-mediated decay (NMD) pathway (15,16). NMD is a widespread and evolutionarily conserved surveillance system that removes messenger RNA (mRNAs) with premature termination codons (PTCs). More importantly, NMD provides a pervasive post-transcriptional mechanism of gene expression regulation, called unproductive splicing, in which PTCs are introduced into transcripts by alternative splicing (AS) to trigger mRNA degradation (17,18). PTCs may appear due to inclusion of the so-called poison exons (PEs) or due to other AS events that induce frameshifts (19,20).

Unproductive splicing shapes cellular transcriptomes in a tissue- and disease-specific manner (18,21). It is a regulated process that depends on RNA-binding proteins (RBPs) and RNA structure, which together orchestrate complex AS programs (22). Many RBPs control their expression via unproductive splicing in an intricate regulatory network with multiple feedback loops (23). Unproductive splicing events often exhibit a remarkable level of evolutionary conservation over long phylogenetic distances (24).

Recently, we characterized a previously unannotated PE in the *BRD3* gene relying solely on evolutionary conservation and evidence from short-read RNA-seq data (25). This example motivated us to elucidate the function of other conserved elements in human introns by taking advantage of the massive amount of RNA-seq data produced by the Genotype-Tissue Expression (GTEx) consortium (26). In contrast to other approaches, which extend the existing AS catalogs departing from transcriptomic data, the method presented here builds on evolutionary conservation and combines it with short-read coverage and split reads to funnel the RNA-seq signal towards putative exons residing in evolutionarily constrained intronic regions.

## Materials and methods

### Cell cultures

Human A549 lung adenocarcinoma cells were maintained in Dulbecco’s modified Eagle’s medium/Nutrient Mixture F-12 with 10% v/v fetal bovine serum (FBS), 1% GlutaMAX (Thermo Fisher Scientific), 0.05 mg/ml streptomycin and 50 units/ml penicillin (all products from Thermo Fisher Scientific) at 37^o^C in 5% CO_2_. Cells were plated at a density of 150 000 cells per well in a 12-well plate. To inactivate NMD, cycloheximide (CHX) was added to the cells 3 h before harvest giving a final concentration of 300 mg/ml in the growth medium. Each experiment was made in at least three independent biological replicates.

### RNA purification and cDNA synthesis

Total RNA was isolated by a guanidinium thiocyanate-phenol-chloroform method using ExtractRNA Reagent (Evrogene) in accordance with the manufacturer’s protocol. One microgram of total RNA was first subjected to RNase-free DNase I digestion (Thermo Fisher Scientific) at 37^o^C for 30 min to remove contaminating genomic DNA. Next, 500 ng of total RNA was used for complementary DNA (cDNA) synthesis using Magnus First Strand cDNA Synthesis Kit (Evrogene) for reverse transcription-quantitative PCR (RT-qPCR) to a final volume of 20 μl. cDNA was diluted 1:5 with nuclease-free water for quantitative PCR (qPCR).

### qPCR

qPCR reactions were performed in triplicates in a final volume of 12 μl in 96-well plates with 420 nM gene-specific primers and 2 μl of cDNA using 5XqPCRmix-HS SYBR reaction mix (Evrogen). Primers for qPCR are listed in Supplementary Table S1. A sample without reverse transcriptase enzyme was included as control to verify the absence of genomic DNA contamination. Amplification of the targets was carried out on CFX96 Real-Time System (Bio-Rad), with following parameters: 95^o^C for 5 min, followed by 39 cycles at 95^o^C for 30 s, 58^o^C for 30 s and 72^o^C for 30 s, ending at 72^o^C for 5 min. Gene and gene isoform expression change was calculated using an estimate of the amplification efficiency value.

### Library preparation and RNA-seq experiments

Illumina cDNA libraries were constructed using NEBNext Ultra II Directional RNA Library Prep Kit for Illumina (New England BioLabs) following the manufacturer’s protocol. Complementary DNA libraries were sequenced using the Novaseq 6000 (Illumina, San Diego, CA, USA) instrument in paired-end mode. In total, ∼37 and 33 million raw reads were obtained for the control and CHX treatment, respectively, with the read length 150 bp. The results of RNA-seq have been deposited at Gene Expression Omnibus under the accession number GSE270310.

### RNA-seq data

The poly(A)+ RNA-seq data obtained from the GTEx project were downloaded in FASTQ format from the dbGaP portal and aligned to the human genome assembly GRCh38 (hg38) using STAR aligner v2.7.8a in paired-end mode (27). GENCODE annotation version 43 was used for the alignment (6). The RNA-seq data from The Cancer Genome Atlas (TCGA) consortium were downloaded from dbGaP portal as pre-computed alignments to the GRCh38 genome assembly in BAM format. The RNA-seq data from RBP perturbation experiments were downloaded from the ENCODE portal also in BAM format (28). For comparative analysis, organ transcriptomes of human, mouse and chicken (hg19, mm10 and GalGal4, respectively) were downloaded from ArrayExpress in BAM format under the accession numbers E-MTAB-6814, E-MTAB-6798 and E-MTAB-6769 (313, 317 and 217 samples, respectively) (29). The results of the experiments on the inactivation of NMD components (UPF1, SMG6, SMG7 and SMG6+SMG7) followed by RNA-seq were obtained from Gene Expression Omnibus under the accession number GSE86148 in FASTQ format and aligned the human genome assembly GRCh38 (hg38) using STAR aligner v2.7.8a in paired-end mode (30).

### The identification of cryptic exons

To identify cryptic exons, transcript assembly and exon expression quantification was performed using StringTie v2.2.1 with --conservative option separately for each sample (31). An exon with coordinates [*x*, *y*] was classified as a cassette exon if its flanking introns [*a*, *x*] and [*y*, *b*] matched an annotated intron [*a*, *b*]. Split reads supporting splice junctions were counted using the IPSA pipeline with the default settings (Shannon entropy of the offset distribution 1.5, canonical GT/AG dinucleotides) (32). Three metrics were assigned to each cassette exon: (i) the average score of the conserved RNA elements UCSC track for 100 vertebrate genomes weighted by the length of the intersection (33), (ii) the mean read coverage reported by StringTie and (3) the split read support by IPSA (the minimum for the two splice junctions). If an exon had more than one pair of flanking introns, the pair with the highest split-read support was chosen. In each sample, cryptic exons were selected on the basis of having the defined metrics exceeding the respective percentiles of the distributions for the annotated cassette exons. The lists of exons were then aggregated across samples and filtered to exclude any sequences overlapping with exons annotated in GENCODE or RefSeq, resulting in a list of cryptic exons. These exons were characterized by their attribution to VastDB (9) and CHESS (8), the percent-spliced-in (Ψ) metric in each RNA-seq sample and the average 100-vertebrate phastCons score (5).

### Exon expression and splicing quantification

The exon inclusion metric Ψ (percent spliced-in, PSI) was calculated as explained earlier (25) using split read counts reported by IPSA that were pooled across all samples in each group (tissue, cancer cohort, RBP depletion experiment, CHX treatment or control) (25). Ψ values with the denominator <40 were discarded. For each cancer type, each RBP inactivation experiment and NMD inactivation, the exon response ΔΨ was calculated between tumors and normal tissues, depletion and control experiments, and CHX treatment and control experiments, respectively.

### Cross-species analysis

The predicted cryptic exons were mapped from the GRCh38 (hg38) human genome assembly to the human (hg19), mouse (mm10) and chicken (galgal4) genome assemblies using CrossMap (34). Only mappings without gaps and with the canonical GT/AG splice sites were used for subsequent analysis. Similarly, the splice sites of the flanking introns were mapped between species, and only introns with the canonical GT/AG dinucleotides were considered. The RNA-Seq data from organ transcriptomes in these species were processed by the IPSA pipeline (34) as before. An exon homolog was considered as expressed in the other species if it was supported by at least one split read at each boundary in at least one sample.

### Conservation analysis of codons

The phyloP conservation scores across 100 vertebrates were downloaded in BigWig format from the UCSC Genome Browser website (33). For exons from the MANE Select dataset, the reading frame was assigned by the annotation. For other exons, the reading frame was inferred by inserting the exon into the MANE Select transcript of the corresponding gene and using the reading frame of the annotated exon following the insertion. To analyze phyloP periodicity independently of the annotation, the average PhyloP score was computed for all three possible phases. The phase with the lowest average PhyloP score in the third position was chosen to assign the reading frame.

## Results

### Re-analysis of GTEx RNA-Seq data reveals cryptic cassette exons

In order to identify cryptic exons, we applied StringTie transcriptome assembly software to short read alignments in each of the 9423 GTEx samples. The resulting transcript models were parsed to identify cassette exons that were flanked by annotated exons both upstream and downstream. Multi-exon skip and other types of AS events were not considered. A cassette exon was classified as cryptic if it was not annotated as an exon in GENCODE or RefSeq and didn’t intersect any annotated exon. Detectable expression levels were observed for 18 085 annotated cassette exons in PC genes and for additional 2310 cryptic exons that were predicted by StringTie.

**Figure 1.**
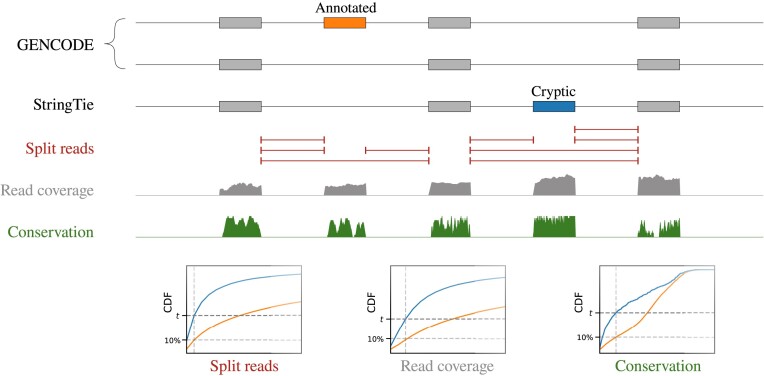
Identification of cryptic exons. Transcript models built by StringTie for each RNA-seq experiment are parsed to identify cryptic cassette exons supported by splice junctions (red), read coverage (gray) and evolutionary conservation score (green). For each of these metrics, the 10th percentile of the distribution for annotated exons is used as a threshold. CDF denotes cumulative distribution function.

**Figure 2.**
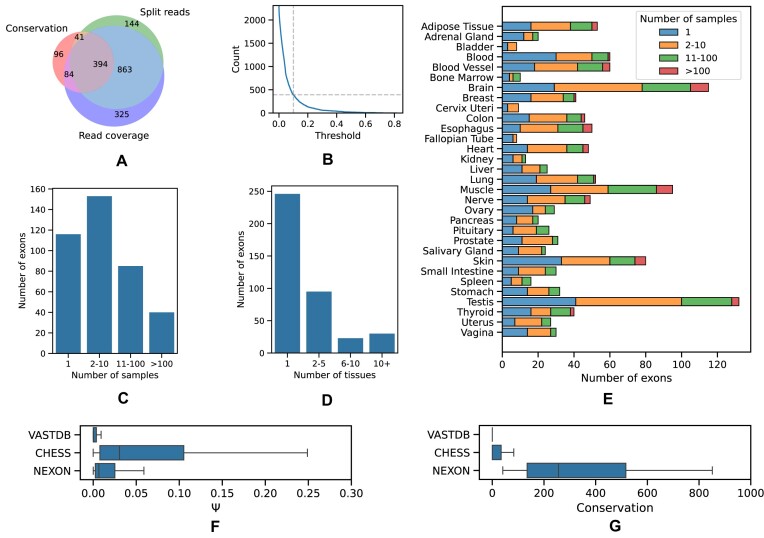
Properties of cryptic exons. (**A**) Venn diagram of the set of cryptic exons passing the 10th percentile threshold by evolutionary conservation, split read support and read coverage support. (**B**) The size of the intersection in (A) as a function of the percentile. (**C**) The support of cryptic exons by the number of samples. (**D**) The support of cryptic exons by the number of tissues. (**E**) The number of exons in each tissue supported by a different number of samples. (**F**) The inclusion levels (Ψ) of exons from VastDB, CHESS and exons reported here (NEXON). (**G**) The average phastCons scores of exons from VastDB, CHESS and NEXON.

The expression level (read coverage per nt), the number of split-reads supporting splice junctions and the conservation score were analyzed further to select sample-specific thresholds (Figure 1). Namely, the 10th percentile of each distribution for the annotated cassette exons was used to select cryptic exons predicted by StringTie with the three metrics exceeding these percentiles. The number of exons defined by each metric alone, their pairwise combinations and all three together indicated that evolutionary conservation was the most restrictive among the three filters (Figure 2A). The intersection consisted of 394 predicted exons (Supplementary File S1) with the median length 94 nts, median coverage of 10 reads per nt, median split-read support of 5 reads and median phastCons conservation score 280 out of 1000 (Supplementary Figure S1). The 10th percentile was chosen as a reasonable midpoint value for the number of predictions decaying as a function of the tail probability (Figure 2B).

The rationale behind selecting sample-specific thresholds was to allow identification of exons that are expressed in rare conditions. Indeed, the distributions of the three metrics varied substantially across samples (Supplementary Figure S2). More than a half of cryptic exons were expressed in <10 samples (Figure 2C), with most exons being detected in just one tissue (Figure 2D). The top-three tissues with the largest number of cryptic exons were brain, testis and muscle (Figure 2E). This imbalance partially reflects the difference in the number of samples per tissue in the GTEx dataset. However, these were also the tissues, in which the largest number of novel exons had been identified earlier (35).

Next, we projected the boundaries of the predicted exons onto the genome sequences of two other vertebrates, mouse and chicken, and computed how many of them are observed in the respective organ transcriptomes (29). Out of 394 human exons, 183 and 41 had orthologs in mouse and chicken, respectively. Furthermore, 238 (60%), 81 (44%) and 26 (63%) of human, mouse and chicken exons were supported at both boundaries in at least one sample. As the difference between these proportions was not statistically significant (χ^2^-test, *P* = 0.12), we concluded that the orthologs of the predicted exons are, indeed, expressed in other vertebrate species.

A number of studies characterized novel exons by re-analyzing large panels of RNA-seq data (36). The largest of them, which resulted in the creation of the VastDB database, detected and quantified >6000 unannotated AS events across vertebrate cell types, tissue types and developmental stages (9). With the 10th percentile cutoff, 221 out of 394 predicted exons (56%) were also found in VastDB, indicating almost twofold enrichment, while with other cutoffs the proportion of exons that were also listed in VastDB varied from 40% to 80% (Supplementary Figure S3A). Further examination of exons that are common to the StringTie predictions and the VastDB catalog shows that those absent from our predicted set substantially decline in terms of read coverage, splice junction support and, most remarkably, evolutionary conservation (Supplementary Figure S3B–D).

We next explored the intersection of our predicted set with CHESS, a human transcript catalog based on large-scale RNA sequencing experiments (8,37). Out of 394 predicted exons, only 33 were present in CHESS. Exons that were also found in CHESS had on average higher inclusion levels across GTEx tissues (Figure 2F), but substantially lower conservation scores (Figure 2G). This analysis demonstrates that the conservation-based approach gives an orthogonal view on constructing exon catalogs and identifies exons that escape RNA-seq screens due to low expression.

### Most cryptic cassette exons are poison exons

**Figure 3.**
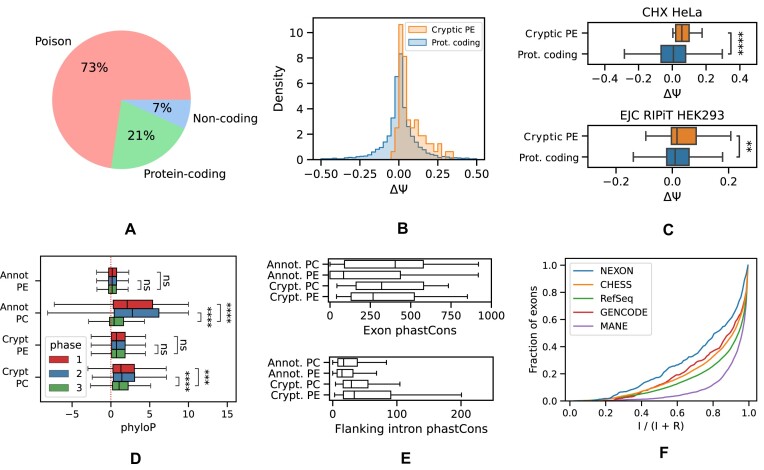
Cryptic exons are mostly poison. (**A**) The proportion of poison, PC and non-coding exons with respect to the MANE Select transcript isoform of the gene. (**B**) The response (ΔΨ = Ψ_CHX_ − Ψ_CTL_) of cryptic PEs and annotated PC cassette exons to NMD inhibition by CHX. (**C**) Same as in (B) but in Hela cell line (43) (top) and for ΔΨ = Ψ_EJC_ − Ψ_RNA − seq_ in the RIPiT experiment (44) (bottom). (**D**) The phyloP scores of annotated and cryptic PEs and PC exons in the first, second and the third codon position (phase 1, 2 and 3, respectively). (**E**) The average phastCons scores of annotated and cryptic poison and PC exons (top) and those of their respective flanking introns (bottom). (**F**) The splicing index *I*/(*I* + *R*), where *I* and *R* are the number of reads supporting intron splicing and retention, respectively, of introns flanking cryptic exons (NEXON) versus exons from other catalogs. Symbols **, ***, **** and ns. denote statistically discernible differences at the 1%, 0.1%, 0.01% significance level and not significant differences, respectively.

To characterize the relationship between cryptic exons in PC genes and their reading frames, we inserted each exon into the representative MANE Select transcript (Matched Annotation from the NCBI and EMBL-EBI collaborative project) of the corresponding gene and translated the resulting nucleotide sequence into amino acids (38). An exon was classified as poison if it introduced a PTC obeying the so-called 50-nt rule (39). This rule postulates that a stop codon is recognized as a PTC if it is located at least 50 nts upstream of the last exon junction. The 50-nt rule rests on the assumption that NMD is triggered by the interactions between the ribosome and exon junction complexes (EJCs), which remain bound to mRNA after the pioneer round of translation (40,41). We found that more than half (73%) of cryptic exons were poison, 21% translated into a protein sequence without a PTC or with a PTC not obeying the 50-nt rule, and a small fraction (7%) were incompatible with the MANE Select transcript or located in untranslated regions (Figure 3A). As expected, PEs were supported by a smaller number of split reads aligning to splice junctions than were PC exons (Supplementary Figure S4).

To test whether the predicted PEs are indeed NMD targets, we assessed how their inclusion level changes in response to NMD inactivation. Towards this goal, we performed an RNA-seq experiment in A549 cell line, in which the NMD system was inactivated by a selective translation elongation inhibitor CHX (42) (Figure 3B). The median difference in exon inclusion levels between CHX treatment and the untreated control for PEs (ΔΨ) showed a significant departure towards positive values (signed rank test, *P*-value < 10^−7^). Similarly, ΔΨ values for cryptic PEs were on average larger than those for annotated cassette exons (sum of ranks test, *P*-value = 0.005). Examination of a similar RNA-seq experiment with NMD inactivation in the HeLa cell line (43) confirms this observation (Figure 3C, top).

The CHX treatment blocks the elongation phase of eukaryotic translation, thereby inhibiting the NMD pathway, but it also affects downstream processes. This motivated us to analyze other inactivation experiments targeting specific components of the NMD pathway (14). In the depletion of UPF1, SMG6, SMG7 and codepletion of SMG6 and SMG7, we observed 126 cryptic PEs with positive and negative responses, where positive responses were significantly stronger (ΔΨ = 0.02, signed rank test *P* < 10^−5^, Supplementary Figure S5). In the inactivation of UPF1 alone, fewer such exons were observed, but the median response was larger (ΔΨ = 0.025).

Besides NMD inhibition, there are other approaches to detect transcripts that are degraded by NMD, for instance, sequencing of transcripts bound to EJCs. In particular, the RIPiT protocol extracts post-splicing, pre-translational mRNA–protein particles from which RNA is subsequently sequenced (44). To check whether cryptic PEs are supported by RIPiT, we compared the differences in their inclusion rates between EJC pulldown and the reference RNA-seq experiments in HEK293 cell line to those of annotated exons (Figure 3C, bottom). Indeed, the predicted PEs were significantly more included in EJC pulldown libraries (signed rank test, *P*-value = 0.01). Taken together, these observations indicate that most cryptic cassette exons inducing PTCs are, indeed, PEs.

However, MANE Select represents only a small fraction of human transcript isoforms, and it could be that an exon introduces a PTC in one transcript but serves as a coding exon in another. To address this question, we inserted each of the 394 exons into each of the CHESS transcripts. Exons were split into three groups: always poison (APE), always protein-coding (APC) and mixed (MIX), which contained exons that induce a PTC in one but not all transcripts. As expected, the APE group was dominating (262 versus 67 in APC versus 65 in MIX) and consisted mainly of exons with lengths that are not multiples of three. MIX exons had a significantly higher frameshift frequency (64% ± 12%) as compared to APC (34% ± 11%) but not significantly different from that in APE (73% ± 5%). Furthermore, 54 out of 65 MIX exons belonged to 5′-UTRs of short transcripts and didn’t induce PTCs because they were outside of the reading frame. This indicates that exons introducing a PTC into the MANE isoform are also used as PEs in other transcripts.

To further confirm this claim, we analyzed the level of nucleotide sequence conservation in the third position of codons located in poison and PC exons. As one would expect, the phyloP conservation score significantly drops in the third position for PC exons but not for PEs, both cryptic or annotated (Figure 3D). Furthermore, cryptic PEs are more similar to the annotated ones by the magnitude of phyloP scores. Since phase is defined with respect to the transcript, into which exon is inserted, we repeated this analysis choosing the most conservative phase (see ‘Materials and methods’ section). It showed that despite small differences between phases, the phyloP score in all three codon positions of PEs remains at the same low level as in the third codon position of PC exons (Supplementary Figure S6).

In some PEs, the high level of nucleotide sequence conservation extends into the flanking introns (45,46). We checked to what extent it is true for all PEs by comparing the average phastCons scores of poison and PC exons and also those of their flanking introns. By construction, the exons reported here tend to be more conserved than annotated ones, but all PEs are generally less conserved than PC ones (Figure 3E, top). Interestingly, the introns flanking cryptic exons also tend to be more conserved (Figure 3E, bottom). Since such introns were observed in expressed sequence tags (ESTs) as being retained and introducing PTCs (45), we computed the intron splicing index, defined as the number of reads supporting splicing as a fraction of the number of reads supporting splicing and retention, across the GTEx dataset. While flanking intron retention was most frequent among cryptic exons compared to other exon classes, the median splicing index for cryptic exons was ∼80% indicating that a relatively small fraction of introns is retained (Figure 3F). Nevertheless, these findings suggest that intron retention may be a common phenomenon in ultraconserved genomic elements carrying PEs.

### Cryptic exons demonstrate specific expression and regulation patterns

**Figure 4.**
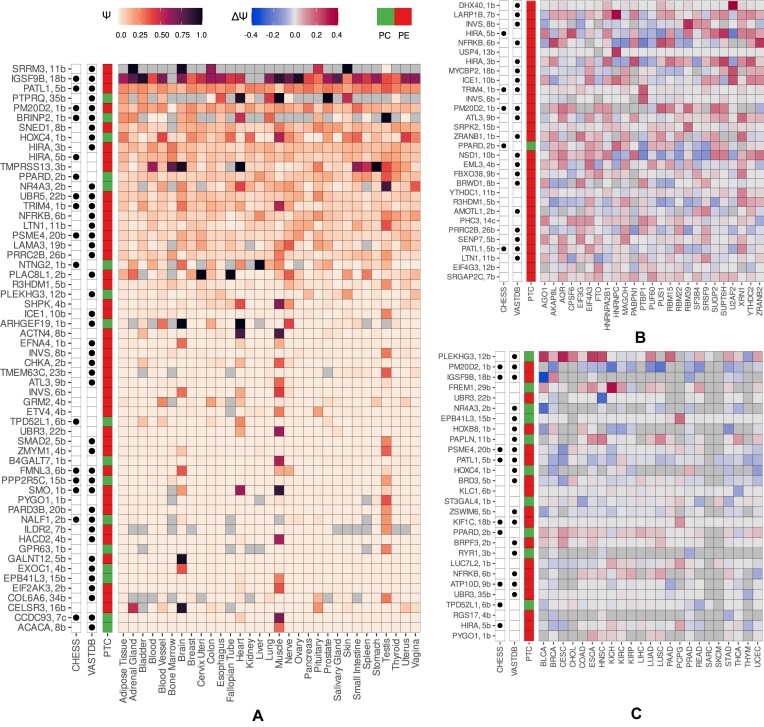
Expression and regulation patterns of cryptic exons. (**A**) The pattern of splicing of cryptic exons across GTEx tissues. Colored squares represent Ψ value. Gray squares represent missing Ψ values. PEs and PC exons, as well as exons present in VastDB and CHESS databases are indicated in the leftmost columns. (**B**) The pattern of splicing changes (ΔΨ = Ψ_KD_ − Ψ_CTL_) in RBP inactivation experiments. (**C**) The pattern of splicing changes (ΔΨ = Ψ_tumor_ − Ψ_normal_) in paired samples from TCGA cohorts.

In order to find conditions in which cryptic exons are expressed, we quantified their inclusion levels (Ψ) across GTEx tissues and selected exons with the median inclusion level of at least 20% in at least one tissue (Figure 4A). According to this criterion, most exons were expressed in only one or in a small number of tissues, with the largest number of tissue-specific exons being observed in the brain, muscle and testis. Remarkably, out of 58 cases satisfying this condition, 41 (71%) were PEs. In some cases, PEs were included in all tissues except a few, suggesting that tissue-specificity is controlled by their skipping.

Next, we assessed the response of cryptic exons to RBP perturbations in a panel of RNA-seq experiments by the ENCODE consortium (28). In doing so, we focused on exons that change the level of inclusion upon RBP inactivation by at least 10% and selected RBPs that supposedly regulate (|ΔΨ| ≥ 0.05) more than ten such exons (Figure 4B). This filter yielded 30 exons, all but one of which were poison, reflecting the predominance of NMD targets among regulated cryptic exons (Fisher’s exact test, *P*-value = 0.01). In most cases, the value of ΔΨ under RBP inactivation was positive, confirming that cryptic exons are normally suppressed by RBPs (signed rank test, *P*-value < 10^−5^, Supplementary Figure S7).

In applying the same analysis and |ΔΨ| ≥ 0.05 threshold to the TCGA RNA-seq panel, we observed cryptic exons with both positive and negative changes in cancer vs. normal tissue (Figure 4C). However, unlike findings in RBP perturbation experiments, there were a few events specific for distinct cancer types, and there was no enrichment of PEs. This observation reflects a more complex landscape of deregulation of AS programs in cancers as compared to inactivation of a single RBP, particularly in genes with known cancer-associated functions (47), two of which are discussed below.

One is the *PRRC2B* gene, which encodes an RBP that regulates genes important for cell cycle progression and proliferation (48,49). Members of the proline rich coiled-coil 2 (PRRC2) protein family, to which it belongs, are associated with immune infiltration and immune escape in hepatocellular carcinoma and undergo AS in primary non-small cell lung tumors (50,51). *PRRC2B* is highly expressed in many tumors, and its overexpression is associated with poor prognosis (48,52). The other gene is *PATL1* (also known as *PAT1b*), which has been identified as a prognostic factor for nasal-type natural killer/T-cell lymphoma and head and neck squamous cell carcinoma (53). It encodes an RBP participating in mRNA decay and AS regulation, where it promotes the inclusion of multiple cassette exons by interacting with tri-snRNP (54).

We found that *PRRC2B* and *PATL1* contain cryptic PEs, which strongly respond to NMD inactivation by CHX (ΔΨ = 0.12 and ΔΨ = 0.27, respectively). The examination of matched-pair RNA-seq samples from TCGA hepatocellular carcinoma cohort (*n* = 50) revealed a small by absolute value but not statistically significant increase in PE skipping (the mean ΔΨ = −0.013, P = 0.06). A similar examination of head and neck squamous cell carcinoma paired samples (*n* = 43) revealed a significant increase in *PATL1* PE skipping (the mean ΔΨ = −0.03, P = 0.02). Remarkably, skipping of these exons also increased (ΔΨ < 0) in 70% of the other TCGA cohorts studied. According to Figure 4B, *PRRC2B* and *PATL1* are regulated by multiple RBPs, however the exact mechanism of regulation remains open to future studies. Here, we select these and a few other cases to experimentally validate them for being PEs.

### Experimental validation of poison exons

When choosing candidates for experimental validation, we required that cryptic exons changed their inclusion rate by at least 0.05, the host gene expression levels increased at least fourfold, and exon boundaries were supported by at least 10 split reads according to the RNA-seq experiment with CHX treatment. Furthermore, we focused on genes that are associated with cancers or known to be regulated by cancer-associated RBPs and gave preference to exons that were also present in VastDB.

Eventually, we chose cryptic exons in eight candidate genes: *SENP7*, *SPAG9*, *PATL1*, *UBR5*, *PRRC2B*, *NSD1*, *SMAD2* and *INVS*. Two of them, *PATL1* and *PRRC2B* were discussed earlier; *SENP7* is a marker of poor prognosis in colon cancer (55); *SPAG9* is expressed in a variety of malignancies and regulated by QKI in lung cancer (56); *UBR5* promotes postsurgical breast cancer lung metastases (57), and *NSD1* exerts tumor suppressive functions (58).

**Figure 5.**
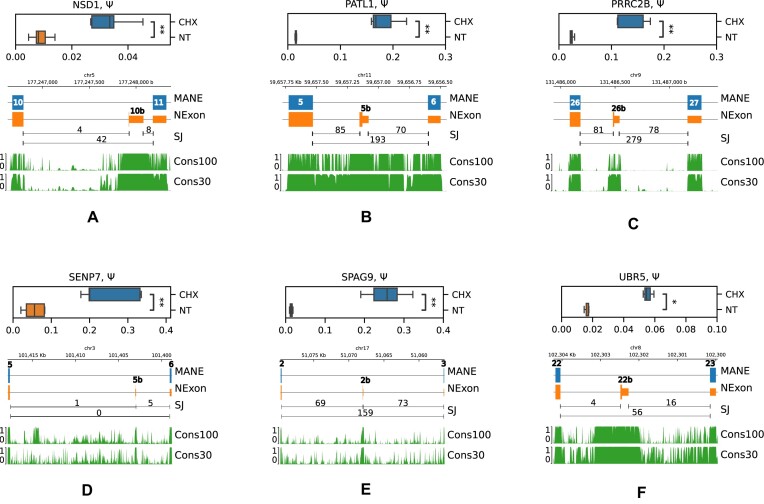
RT-qPCR validation of cryptic PEs in NSD1 (**A**), PATL1 (**B**), PRRC2B (**C**), SENP7 (**D**), SPAG9 (**E**) and UBR5 (**F**) genes. Boxplots represent Ψ values across five biological replicates. The SJ track shows the number of split reads supporting splice junctions in NMD inactivation by CHX. Symbols * and ** denote statistically discernible differences at the 5% and 1% significance level. The diagram below shows the MANE Select PC isoform with exon numbers, the unproductive isoform (NEXON) and conservation scores (from 0 to 1) across 100 vertebrate species (Cons100) and 30 placental mammals (Cons30).

To assess splicing changes in these genes, we designed qPCR primers that allow differential measurements of splice isoforms, in which the cryptic exon is included or skipped (Supplementary Table S1). Out of eight exons tested, seven showed a significant upregulation upon NMD inactivation by CHX (Figure 5 and Supplementary Figure S8A) and exon 6b in *INVS* showed an insignificant change in the opposite direction (Supplementary Figure S8B). While in some genes (*PATL1*, *PRRC2B*, *SENP7* and *SPAG9*), the magnitude of splicing changes was comparable with ΔΨ values from the RNA-seq CHX experiment; in others (*UBR5* and *NSD1*), it was relatively small and amounted only to 2%. However, all these genes were selected on the basis of having at least 4-fold increase in the expression level under CHX treatment. Therefore, despite small absolute values of ΔΨ, the fold change with respect to the baseline expression level was substantial. This discrepancy in *UBR5* and *NSD1* may be attributed to concurrent intron retention considering that evolutionarily conserved regions extend beyond PEs in the alignments of 100 vertebrates and 30 mammals (Figure 5).

### Protein-coding cryptic exons

Despite most cryptic exons being poison, a small fraction were in frame with respect to the main PC isoform. Since it is rather unusual to discover novel coding elements in the vastly annotated human genome, we looked further into the amino acid sequences of these exons. The average probability of disorder (pD) for amino acid residues in PC cryptic exons, which was computed using ODiNPred tool (59), didn’t significantly differ from that of adjacent PC exons (Wilcoxon test, *P*-value = 0.77). In application of InterProScan (60), only four exons contained disordered regions predicted by MobiDB-lite, and no other features were detected, indicating that cryptic exons do not introduce additional protein domains.

To assess the similarity of cryptic PC exons to the existing proteins, we searched their amino acid sequences in the human and mouse RefSeq curated protein databases using blastp with *E*-value cutoff of 10^−5^. When searching against the human database, the only similarity was found between the exons 4b and 4c in the *PLAAT3* gene and a short stretch of amino acids in the *TNPO3* gene. When searching against the mouse database, exons 12b in *PLEKHG3* and 4b in *CAMSAP1* were aligned to the homologous sequences in mouse (Plekhg3 and Camsap1) with the same ordinal exon numbers (13 and 5, respectively). Since full characterization of the impact exerted by PC cryptic exons on protein structure is far beyond the scope of this study, we chose not to investigate them further.

## Discussion

In the era of high-throughput sequencing, the genome annotation has become increasingly accurate but also increasingly complex. High-fidelity annotation requires multiple levels of evidence, such as presence of a specific protein, expression of the mRNA and the presence of the corresponding locus in the genome. Most transcriptomic studies describe splice isoforms based only on RNA-seq evidence, although the extent of the functional impact of AS remains a matter of debate (61–65). A recent study suggested that much of the impact of AS is mediated by the generation of NMD isoforms to control gene expression rather than diversification of the proteome (66).

Numerous reports have indicated a tremendous splicing variability among cells, tissues, developmental stages and individuals (67,68). It is, therefore, challenging to distinguish between functional transcript isoforms and splicing noise (69). Noisy transcripts often have low abundance due to pervasive, low-level transcription, but their low expression may also be a result of high transcript degradation rate, as it is the case for NMD targets. Consequently, unproductive splice isoforms are generally underrepresented in RNA-seq libraries (44). Yet, unproductive splicing exerts global influence on gene expression profiles (66) and plays an essential role in the onset and progression of many diseases, particularly in oncogenic repression or stabilization (70,71). In serine/arginine-rich (SR) proteins, unproductive splicing is coordinated by a large network of PEs, which simultaneously control the expression levels of many genes (46).

Our study confirms that the majority of the newfound cassette exons are poison, and they react accordingly to NMD inhibition by CHX and are enriched in EJCs. They show a broad pattern of tissue-specificity, cancer-specificity and regulation by RBPs in accordance with many previous reports (21,72–74). Some exons are regulated by a large number of RBPs, while others have a unique regulator, yet many are controlled by RBPs with known splicing regulation activity such as hnRNPs and SR-rich factors. These observations indicate that the newfound exons represent a legitimate class of transcript elements, some of which were overlooked in databases, and that they complement and extend the existing exon catalogs such as VastDB and CHESS.

The methodology presented here builds on nucleotide sequence conservation as a primary feature to detect cryptic exons. It is most effective for discovering cassette exons residing in standalone intronic elements, but it can also be applied to other AS types such as retained introns or alternative splice sites. In the latter case, however, the evolutionary conservation of intronic sequences adjacent to exons may be not as discriminatory to distinguish between functional splice sites and splicing noise. Tandem alternative splice sites represent a category of splicing events that is particularly difficult to detect using evolutionary conservation alone (75).

Approximately three quarters of cryptic exons described here are PEs, confirming that evolutionary conservation is a hallmark of unproductive splicing (24). Quite frequently one can see not only the nucleotide sequence of the PE but also the nucleotide sequences of its flanking introns being highly conserved. In fact, PEs were first discovered based on this very feature (19). Examples of extreme intronic sequence conservation can be found in genes encoding SR proteins such as *SRSF3* and *SRSF7* (76,77), but in both these cases are relatively short. The results obtained here indicate that the conservation of the intron as a whole is not a universal feature of all PEs, and that it may be characteristic to some but not all PEs.

In total, the newfound cryptic exons encompass 45 441 nucleotides, which represents only a tiny fraction (∼0.1%) of the conserved intronic sequence. While it appears plausible that more cryptic exons may be discovered in application of the described method to larger RNA-seq panels, it is quite clear that not all of the conserved intronic sequences represent functional exons. According to our estimates, ∼0.2% of conserved intronic nucleotides belong to cassette exons listed in VastDB and, conversely, only 4% of nucleotides occupied by VastDB cassette exons are conserved across 100 vertebrates. Other functional and, therefore, conserved intronic sequences may serve as transcription factor binding sites, RBP-binding sites (78,79) or RNA structural elements (80,81).

RNA structure has emerged as a critical factor in AS regulation, in which intronic base-pairings loop out exons and cause their skipping (81,82). Earlier we reported that the vertebrate *BRD2* and *BRD3* genes independently evolved flanking RNA structures to control PE skipping (25). RNA *in situ* conformation sequencing indicates that PEs generally tend to be flanked by RNA structures (80). Here, we observed a broad tendency for cryptic PEs to be surrounded by pairs of conserved complementary regions (PCCRs) (81): 25% of cryptic PEs had at least one flanking PCCR, while only 13% of annotated cassette exons did so. However, due to the lack of statistical differences between structure energies and confirmation by probing assays, we didn’t pursue this analysis any further.

Evolutionary conservation has long been used by biologists to discover functional genomic elements. Nucleotide sequence conservation is often combined with other phylogenetic features such as coding potential to identify novel exons in PC genes. However, as we demonstrate here, human introns contain a large number of cryptic PEs, which serve regulatory purposes rather than encode amino acids. While here we studied sequences that are conserved across 100 vertebrate genomes, considering shorter evolutionary spans must further extend the catalog of NMD targets. Therefore, future studies should re-examine introns of human genes taking into account evolutionary conservation to construct phylogenetically supported transcript models.

## Supplementary Material

lqae163_Supplemental_Files
