# Supplementary material for: Human introns contain conserved tissue-specific cryptic poison exons
Source: NAR Genom Bioinform. 2024 Dec 11;6(4):lqae163. doi: 10.1093/nargab/lqae163 (PMC11632617; doi:10.1093/nargab/lqae163)
Supplement: lqae163_Supplemental_Files [file lqae163_supplemental_files.zip › nexon_supp.pdf]

SUPPLEMENTARY INFORMATION: Human introns contain conserved tissue-specific cryptic poison exons

Sergey Margasyuk[1,†], Antonina Kuznetsova[1,†], Lev Zavileyskiy[1], Maria Vlasenok[1], Dmitry Skvortsov[1,2], and Dmitri D. Pervouchine[1,*]

[1]Skolkovo Institute of Science and Technology, Moscow 121205, Russia

[2]Moscow State University, Faculty of Chemistry, Moscow 119991, Russia

[†]These authos contributed equally to this work

[*]e-mail: d.pervouchine@skoltech.ru; tel/fax +7 (495) 280 14 81

October 10, 2024

List of Figures

S1 The distributions of lengths, read coverage, the number of supporting split reads, and average phastCons conservation score for cryptic exons 3

S2 Variability in the distributions of cryptic exons metrics 4

S3 Comparison of cryptic exons to VastDB exons 5

S4 The number of split reads supporting poison and protein-coding cryptic exons . 6

S5 The response to UPF1, SMG6, SMG7 and SMG6+SMG7 knockdowns 7

S6 The phyloP scores computed using a conservative coding frame choice 8

S7 The distribution of $\Delta\Psi$ values in RBP inactivation experiments 9

S8 RT-qPCR validation of cryptic poison exons in SMAD2 and INVS genes 10

List of Tables

S1 RT-qPCR primers 12

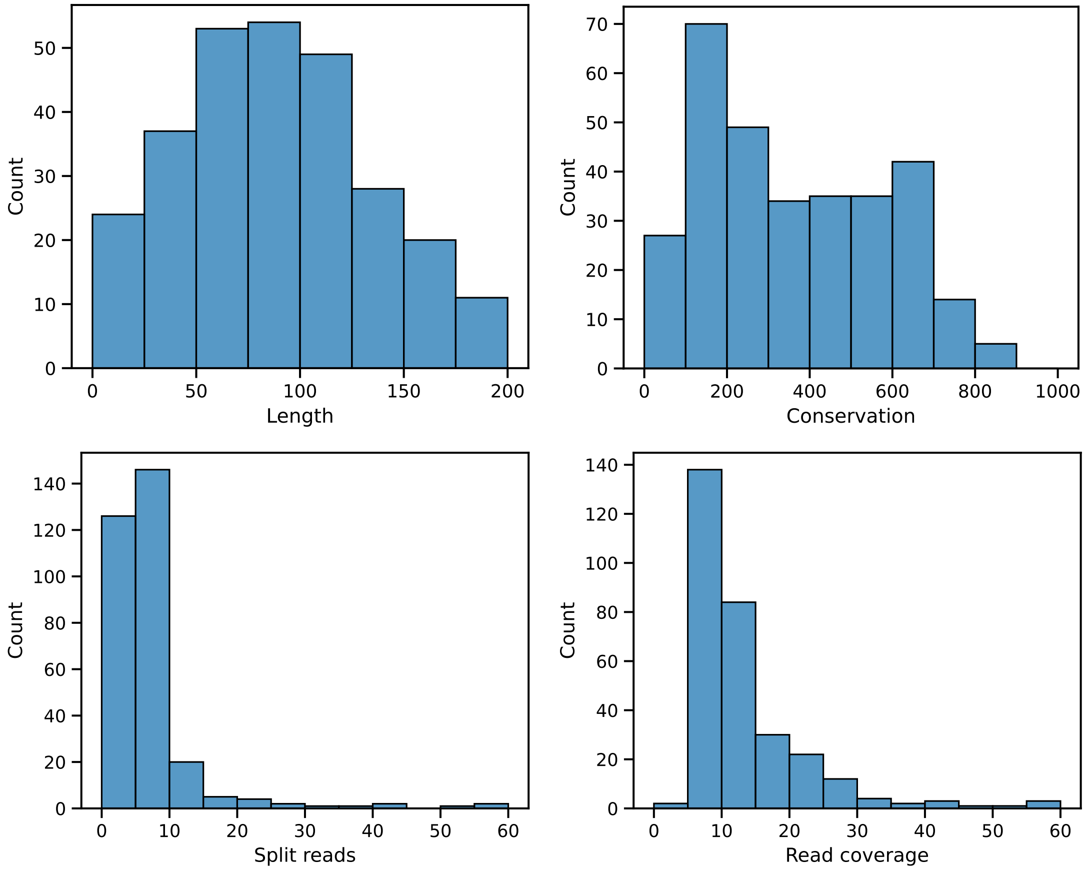

Figure S1: The distributions of lengths (nts), read coverage (reads per nt), the number of split reads supporting splice junctions, and average phastCons conservation score for cryptic cassette exons.

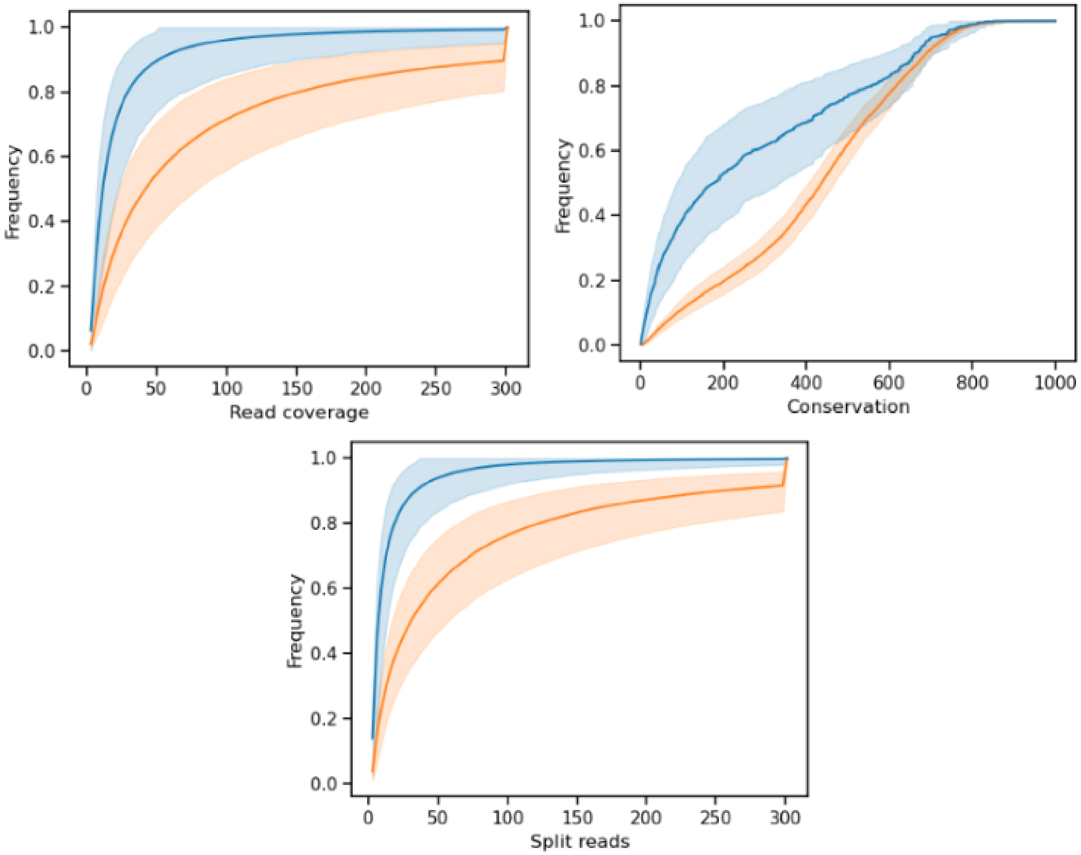

Figure S2: Variability in the distributions of the three metrics (the read coverage, the phastCons conservation score, and the number of split reads supporting splice junctions) across GTEx samples for annotated (yellow) and cryptic (blue) cassette exons. The median distribution is shown by a thick curve.

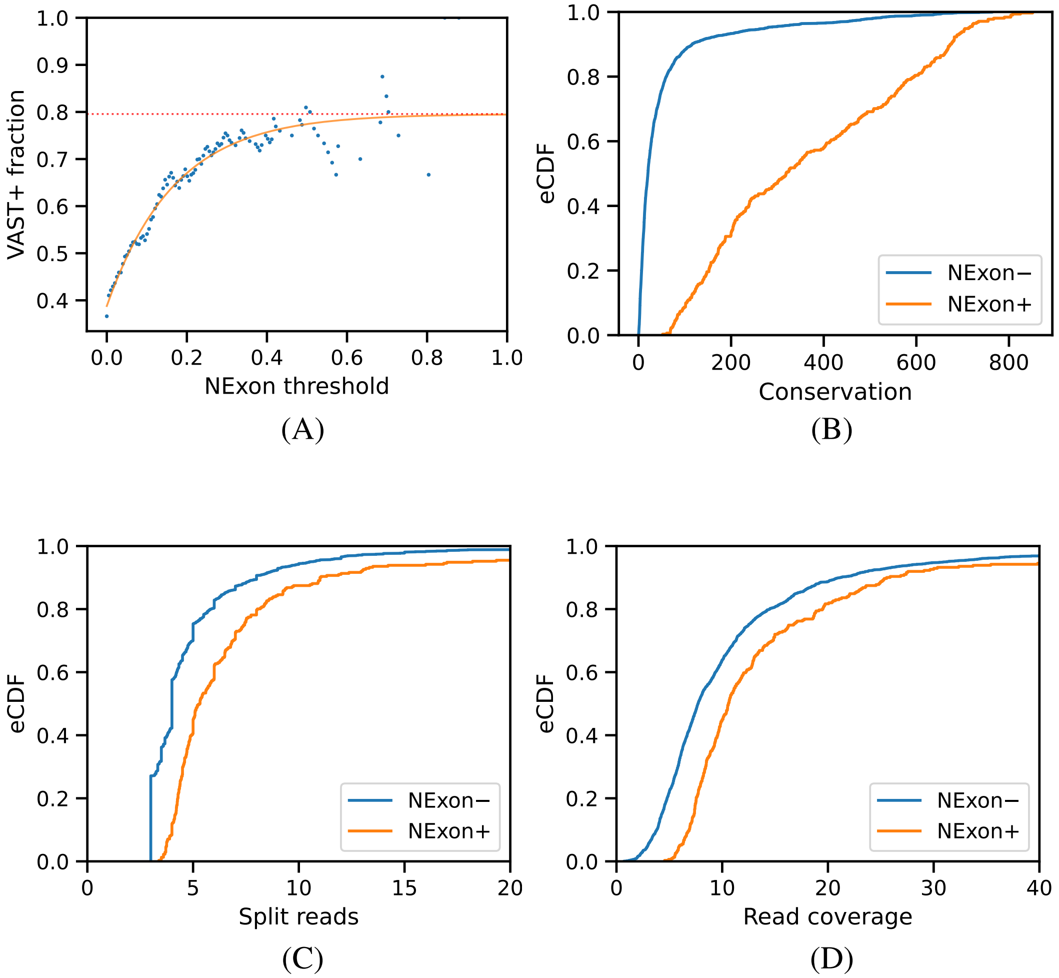

Figure S3: Comparison of cryptic exons to VastDB exons. **(A)** The proportion of predicted exons that are also listed in VastDB as a function of percentile cutoff. **(B)** The cumulative distribution function (eCDF) of phastCons scores of VastDB exons that passed the 10th percentile cutoff (NExon+) vs. the remaining exons (NExon−). **(C)** and **(D)** are same as (B) for the number of supporting split reads and the read coverage, respectively.

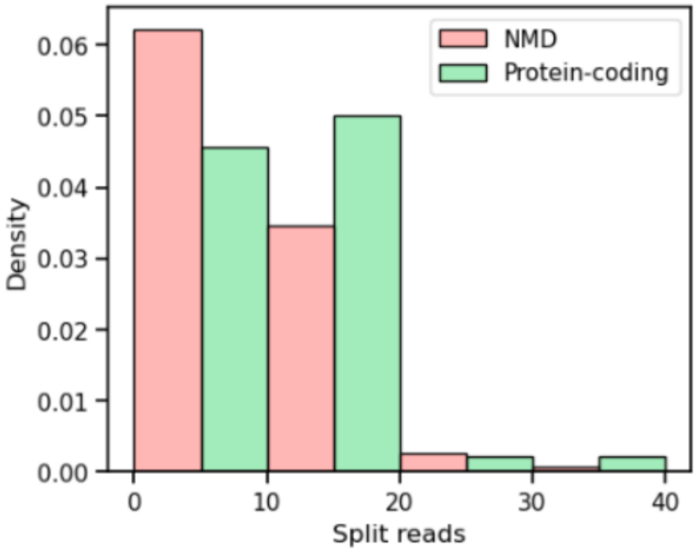

Figure S4: The number of split reads supporting poison (NMD) and protein-coding cryptic exons.

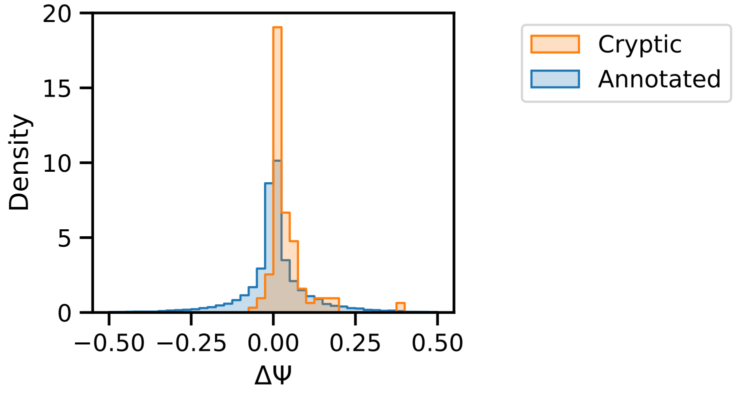

Figure S5: The response ($\Delta\Psi = \Psi_{CHX} - \Psi_{CTL}$) of cryptic poison exons and annotated protein-coding cassette exons to NMD inhibition by UPF1, SMG6, SMG7 and SMG6+SMG7 knockdowns (pooled).

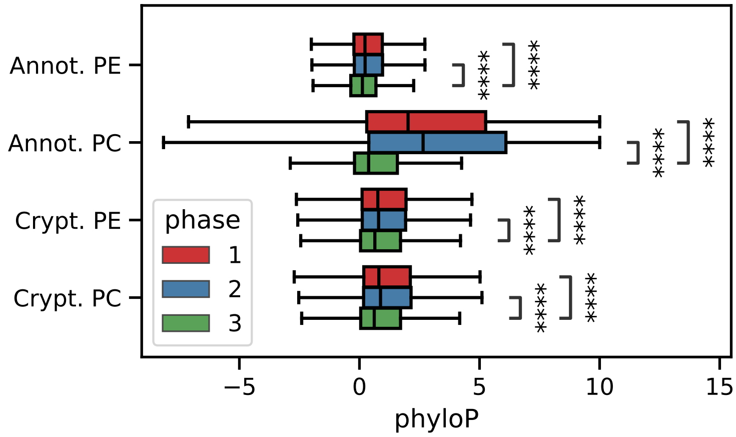

Figure S6: The phyloP scores of annotated and cryptic poison exons (PE) and protein-coding (PC) exons in the first, second, and the third codon position (phase 1, 2, and 3, respectively) using a conservative coding frame choice.

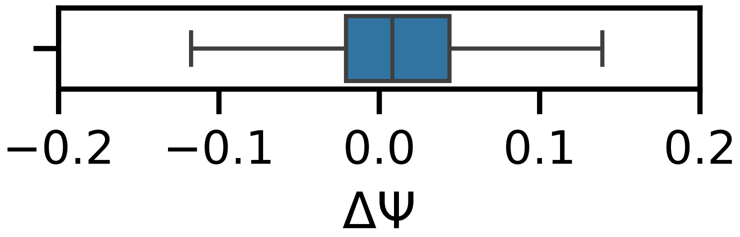

Figure S7: The distribution of $\Delta\Psi = \Psi_{KD} - \Psi_{CTL}$ values in RBP inactivation experiments (Figure 4B).

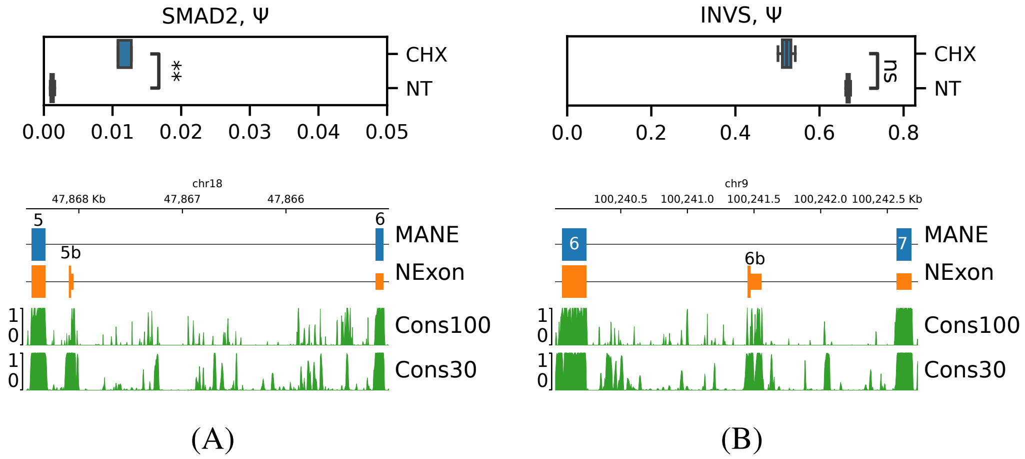

Figure S8: RT-qPCR validation of cryptic poison exons in SMAD2 (A) and INVS genes (B). Changes in *SMAD2* exon 5b are statistically significant at the 1% significance level, however small by absolute value. Changes in *INVS* exon 6b are not significant and go in the opposite direction.

SupplementaryDataFile 1: The list of conserved cryptic exons and their attributes

Primer ID	Primer sequence
PATL1_F	GCGGTCAACTTCACCTATCA
PATL1_R	GCTTCGGACACAGAATCTGT
PATL1_5bR	GGACTGCACAGGCTCTGT
UBR5_F	CATCAAGTAGCCGAAGTTTGAGAT
UBR5_R	TGGTGGTCACTGGATGAAGC
UBR5_22bF	CAGCCACTGAGGATACAGTTTGA
SMAD2_F	AGAACTTCCGCCTCTGGAT
SMAD2_R	GAGGTGGCGTTTCTGGAAT
SMAD2_5bR	CAGGAGGTGGCGTTTTAAGAT
SENP7_F	TGATGCCAACAAGGTGCAAT
SENP7_R	CGTCAATATAACTCTGGGTATCCTT
SENP7_5bR	TTCATCATTCTAGATGCCTCTTTCA
SPAG9_F	ACAGACCCGAGTGGAATCTT
SPAG9_R	TCTTTCTTCAAGTCTGCTAATCTGG
SPAG9_2bR	TCAGACTGCTGATAGGCTCTG
PRRC2B_F	GCAGAGTTACCAACAGGCCG
PRRC2B_R	GATCTCCTGGGACTGGGACA
PRRC2B_26bF	TGGATGCATTAGAAAGAGGCCG
NSD1_F	TGACAAGCCAAGGAAGCGAA
NSD1_R	GGCATTAGTTCTCCCTCTGAGC
NSD1_10bR	TCCATTTTAAGGTTTCACTCTGAGC
INVS_F	CTGGGCAGCTTTATTAGGCCAT
INVS_R	GTCAGATGGGATAGTTCCAGACTT
INVS_6bF	AGATCATTTTCAGAGGCCATGC

Table S1: RT-qPCR primers.
